# Emotional stress as a trigger of myasthenic crisis and concomitant takotsubo cardiomyopathy: a case report

**DOI:** 10.1186/1752-1947-4-393

**Published:** 2010-12-03

**Authors:** Said R Beydoun, JingTian Wang, Reed Loring Levine, Ali Farvid

**Affiliations:** 1 Department of Neurology, University of Southern California, Los Angeles, California, USA; 2 Department of Anesthesiology, University of Southern California, Los Angeles, California, USA; 3 Department of Cardiology, University of Southern California, Los Angeles, California, USA

## Abstract

**Introduction:**

Myasthenia gravis is a neuromuscular junction post-synaptic autoimmune disorder. Myasthenic crisis is characterized by respiratory failure requiring mechanical ventilation. Takotsubo cardiomyopathy is a rare clinical syndrome defined as a profound but reversible left ventricular dysfunction in the absence of coronary artery disease.

**Case presentation:**

We report a unique case of a 60-year-old Hispanic woman with myasthenia gravis who developed takotsubo cardiomyopathy and concomitant myasthenic crisis that appear to have been triggered by a stressful life event. On admission, she presented with severe mid-sternal chest pain and shortness of breath shortly after a personally significant stressful life event. A pertinent neurological examination showed bilateral facial weakness and right ptosis. The left ventriculogram showed apical ballooning with hyperdynamic proximal segments with sparing of the apex. Her troponin I level was elevated, while cardiac catheterization revealed no significant coronary artery disease. The findings were consistent with takotsubo cardiomyopathy. Shortly after cardiac catheterization, she developed bilateral ophthalmoparesis and significant bulbar and respiratory muscle weakness. Forced vital capacity values were persistently less than 1 L. The patient developed respiratory failure and required endotracheal intubation. After plasmapheresis and corticosteroid treatment, her clinical course improved with successful extubation. A normal left ventricle chamber size and a normal ejection fraction were noted by an echocardiogram repeated 10 months later.

**Conclusion:**

This is the first reported case of the simultaneous triggering of both takotsubo cardiomyopathy and myasthenic crisis by the physiologic consequences of a state of severe emotional stress. We hypothesize that the mechanism underlying the rare association of takotsubo cardiomyopathy with myasthenic crisis involves excessive endogenous glucocorticoid release, a high-catecholamine state, or a combination of both. We advocate careful cardiac monitoring of myasthenia gravis patients during acute emotional or physical stress, as there is potential risk of developing takotsubo cardiomyopathy.

## Introduction

Myasthenia gravis (MG), the most common disorder of the neuromuscular junction (NMJ), is a post-synaptic autoimmune disease. Until recent decades, MG was often fatal, with mortality rates for myasthenic crisis (i.e., respiratory failure requiring mechanical ventilation), which affects up to 20% of myasthenic patients at some point in their illness, as high as 30% to 70% in the early 1960 s [1]. Owing to improved critical care assessment and management, the mortality rate for mysathenic crisis dropped dramatically to about 4% to 8% [2]. Etiologies of myasthenic crisis include infection (one-third or more of cases); aspiration (about 10% of cases), medication change, emotional or physical stress (e.g., surgery, psychological trauma); and in one-third of patients, no clear precipitant is identified [3]. We present a case of takotsubo cardiomyopathy (TC) that appears to have been related to a stressful life event that was believed to have triggered a concomitant MG crisis. TC is a rare but increasingly recognized clinical syndrome characterized by transient left ventricular dysfunction in the absence of coronary artery disease and minimal cardiac enzyme release [4]. To the best of our knowledge, this is the first report delineating the simultaneous triggering of both TC and MG crisis by acute emotional stress. We hypothesize that the mechanism underlying this rare association of TC with MG crisis involves excessive endogenous steroid release, a high-catecholamine state, or a combination of both.

## Case presentation

A 60-year-old Hispanic woman was diagnosed with generalized MG. Her clinical symptoms included right ptosis, diplopia, dysarthria, dysphagia, and muscle weakness that had progressed for three months. Her laboratory workup showed an elevated acetylcholine receptor binding antibody titer of 252.45 nmol/L (normal < 0.30 nmol/L). Repetitive nerve stimulation at a frequency of 2 Hz showed a 48% decremental response of the median nerve compound muscle action potential (CMAP) amplitude, supporting a post-synaptic neuromuscular junction dysfunction. Striational muscle antibody titers were negative, and computed tomography of the chest revealed the absence of anterior mediastinal mass. Her treating neurologist gave her pyridostigmine and mycophenolate mofetil, and she was discharged after improvement of her myasthenic symptoms.

**Figure 1:**
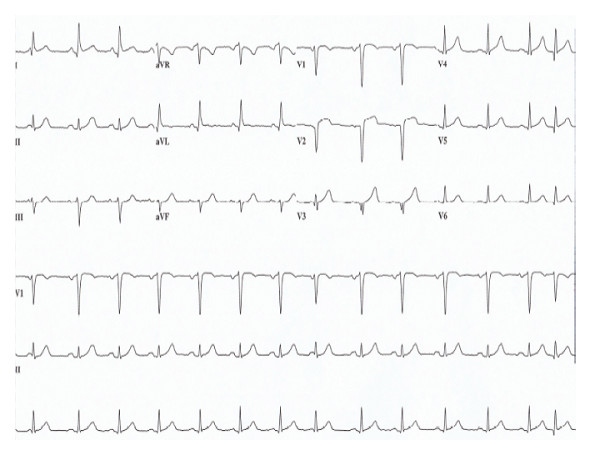
**Electrocardiogram demonstrating sinus rhythm with 2 mm ST elevations in V2 and V3 leads with q-waves**.

**Figure 2:**
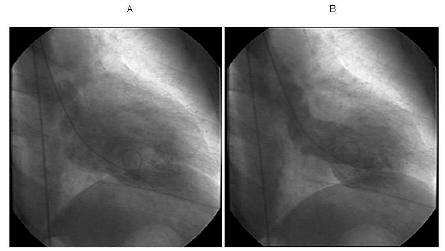
**Left ventriculogram**. **(a) **The diastolic phase. **(b) **The systolic phase showing apical ballooning with sparing of the apex with hyperdynamic basal segments, consistent with takotsubo cardiomyopathy.

One month following the diagnosis of MG, she presented to our emergency department with severe mid-sternal chest pain and shortness of breath shortly after a personally significant stressful life event. On admission, she appeared to be in mild distress. Pertinent neurological findings showed bilateral facial weakness and right ptosis. Her troponin I level was elevated at 2.5 ng/ml (normal: < 0.1 ng/ml). Her electrocardiogram (ECG) on presentation showed sinus rhythm with 2 mm ST elevations in V2 and V3 leads with q-waves (Figure 1). Cardiac catheterization revealed no significant coronary artery disease. The left ventriculogram (Figure 2) showed apical ballooning with hyperdynamic proximal segments with sparing of the apex. The pattern of wall motion abnormality did not fit any single coronary artery distribution and was consistent with TC, also known as stress cardiomyopathy or broken heart syndrome. Left ventricular ejection fraction (LVEF) was calculated as 32% on a cardiac catheterization and 40% on a transthoracic echocardiogram.

Shortly after cardiac catheterization, she developed bilateral ophthalmoparesis, significant bulbar muscle weakness with worsening dysphagia and dysarthria, respiratory muscle weakness with hypoventilation and generalized weakness, and evolving respiratory failure requiring endotracheal intubation. She underwent six plasmapheresis sessions on an every other day regimen. She was started on prednisone 20 mg daily with gradual titration to 60 mg daily. Pyridostigmine dosage was adjusted and titrated slowly. Her clinical course improved gradually, and at the end of her 17-day hospitalization, she was weaned off the ventilator and later discharged home.

She has been regularly followed by our neuromuscular service since discharge. Her myasthenic symptoms continued to improve requiring a low dosage of pyridostigmine and prednisone tapered slowly to a minimal every other day dosage of 10 mg. She also has been seen by the cardiology service, where a repeat echocardiogram 10 months later revealed a normal left ventricular chamber size and a normal ejection fraction.

## Discussion

This report described a rare association of TC with MG crisis. On the basis of a MEDLINE search, two case reports were found in the literature addressing the association of TC with MG crisis. Both patients in those reports were initially admitted for MG crisis, without any identified psychological stress, and subsequently developed TC following plasmapheresis treatments. Both reports suggested that the MG crisis itself and/or plasmapheresis treatments played a causative role in the development of TC [5,6]. In contrast, our patient with a recent diagnosis of MG presented with TC after major emotional stress which was shortly followed by MG crisis. In our case, it seems likely that both TC and MG crisis were triggered by the physiologic consequences of a state of severe emotional stress. Given the worsening of our patient's myasthenic symptoms post-cardiac catheterization, it is plausible that the additional stress from the cardiac catheterization precipitated or contributed to the MG crisis. Despite different clinical presentations in the three cases, we hypothesize that there are common underlying pathophysiological mechanisms involving the hypothalamic-pituitary-adrenal axis and catecholamine system.

TC, also called transient left ventricular apical ballooning, is an increasingly recognized clinical syndrome characterized by a profound but reversible left ventricular dysfunction. The word "takotsubo" is Japanese for a fishing pot with a narrow neck and wide base used to trap octopus, and it is used to delineate the shape of left ventricle in dysfunction on ventriculography [7]. TC is also known by many other names, including apical ballooning cardiomyopathy, stress-induced cardiomyopathy, ampulla cardiomyopathy, and broken heart syndrome. It is a clinical diagnosis generally defined as an acute left ventricular systolic dysfunction with apical ballooning of unknown etiology that is associated with emotional or physical stress without significant obstructive coronary artery disease to account for the clinical findings. Its symptoms and associated ECG results can mimic acute coronary syndromes [8]. Our patient had sparing of the apex, which is a variant of the classically described entity. Treatment of TC is generally supportive. Most patients have an uneventful recovery with a very favorable prognosis. On the basis of a literature review, in-hospital mortality was only 1.1% with only a 3.5% recurrence rate during a wide follow-up period ranging from eight days to four years [4].

There are several proposed pathophysiological mechanisms underlying TC, including multiple simultaneous coronary artery spasms, abnormal perfusion through the microcirculation of the heart, and elevated catecholamine states [4,7]. TC has been described in many different stressful and catastrophic circumstances such as car accidents, family deaths, and even major earthquakes. In our patient, the extremely stressful setting of the sudden death of her cherished bird likely brought on a sudden, very large increase in catecholamine levels and is likely the culprit mechanism. Unique to this case is the concomitant MG exacerbation. It is well known that emotional stress can cause MG crisis by exaggerating or unmasking existing MG symptoms. In line with this view, we hypothesize that emotional stress is directly linked to the mechanism underlying this rare association of TC with MG crisis.

Ample evidence suggests that psychological trauma and/or physical stress stimulate the hypothalamic-pituitary-adrenal axis to release corticoid-releasing hormone (CRH), which causes elevated systemic corticosteroids (e.g., glucocorticoids) and a surge in catecholamines [9,10]. Acute stress initially activates inflammatory cascades through acute phase mediators such as interleukin (IL)-4 and C-reactive protein. At the same time, glucocorticoids which are elevated in response to acute stress down-regulate the inflammatory cascades and maintain homeostasis. In chronic stress conditions such as autoimmune disease, however, there is a disturbance in this normal homeostatic regulation, as chronic stress decreases endogenous glucocorticoid levels and potentially may alter glucocorticoid physiologic effects on the immune system [11-13]. In this context of chronic stress-like conditions due to MG, the surge in glucocorticoid levels in response to acute stress may exert a paradoxical effect on inflammatory cascades, leading to an enhanced immune response. This may explain the well-recognized clinical phenomenon of MG patients developing a worsening of myasthenic symptoms and/or the development of myasthenic crisis when given a high dose of exogenous corticosteroids.

In TC, excessive catecholamine levels in response to emotional and/or physical stress have been reported. Measured early, after the stressful event triggering TC, the catecholamine levels are reported to be up to 34 times higher than normal baseline values, which are even significantly higher than those measured in conditions such as myocardial infarction [14,15]. Elevated catecholamine levels are therefore argued to be an essential link between emotional stress and cardiac injury in TC. It has been proposed that excessive catecholamine in response to acute stress is a potential source of oxygen-derived free radicals which adversely affect sodium and calcium transporters. As a result, an excessive trans-sarcolemmal calcium influx would lead to myocyte dysfunction and injury in TC [14]. We believe that this may have contributed to the development of TC in our MG patient. To date, how a sudden surge in circulating catecholamine levels impacts MG exacerbation has yet to be characterized. Further study will be helpful to elucidate mechanisms underlying catecholamine level variation with MG symptoms.

## Conclusion

Alterations in corticosteroid and/or catecholamine in response to acute and chronic stressors are speculated to have played a key role in the development of MG exacerbation and TC in our patient. We advocate careful cardiac monitoring of MG patients during acute emotional or physical stress, as there is a potential risk of developing TC.

## Competing interests

The authors declare that they have no competing interests.

## Authors' contributions

SRB is the neurologist in chief who dealt with and advised on the patient's neuromuscular condition and management and was a major contributor in writing the manuscript. JTW analyzed and interpreted the patient's data and was a major contributor in writing the manuscript. AF is a cardiologist who analyzed and interpreted the patient's data regarding cardiac symptoms and contributed to writing and review of the manuscript. RLL analyzed and interpreted the patient's data relative to MG symptoms and contributed to writing the manuscript. All authors read and approved the final manuscript.

## Consent

Written informed consent was obtained from the patient for publication of this case report and any accompanying images. A copy of the written consent is available for review by the Editor-in-Chief of this journal.
